# Recurrent somatic BRAF insertion (p.V504_R506dup): a tumor marker and a potential therapeutic target in pilocytic astrocytoma

**DOI:** 10.1038/s41388-018-0623-3

**Published:** 2018-12-21

**Authors:** Fida Khater, Sylvie Langlois, Pauline Cassart, Anne-Marie Roy, Mathieu Lajoie, Jasmine Healy, Chantal Richer, Pascal St-Onge, Nelson Piché, Sebastien Perreault, Sonia Cellot, Monia Marzouki, Nada Jabado, Daniel Sinnett

**Affiliations:** 1 Charles-Bruneau Cancer Centre, Division of Hematology-oncology, CHU Sainte-Justine, Montreal, Canada; 2 Department of Pediatrics, Faculty of Medicine, Université de Montreal, Montreal, Canada; 3 Department of Pediatric Surgery, CHU Sainte-Justine, Montreal, Canada; 4 Division of Neurology, CHU Sainte-Justine, Montreal, Canada; 5 Department of Pediatrics, Faculty of Medicine, McGill University, Montreal, Canada

**Keywords:** Biomarkers, Cancer genomics, Cancer genetics, Cancer therapy, CNS cancer

## Abstract

Pilocytic astrocytoma (PA) is emerging as a tumor entity with dysregulated RAS/RAF/MEK/ERK signaling. In this study, we report the identification of a novel recurrent BRAF insertion (p.V504_R506dup) in five PA cases harboring exclusively this somatic tandem duplication. This recurrent alteration leads to an addition of three amino acids in the kinase domain of BRAF and has functional impact on activating MAPK phosphorylation. Importantly, we show that this mutation confers resistance to RAF inhibitors without changing effectiveness while downstream MEK inhibitors remain effective. Our results further emphasize the importance of BRAF alterations in PA and the need to characterize them in a given tumor as this can affect therapeutic strategies and their potential use as tumor marker in molecular diagnostics.

## Introduction

Pilocytic astrocytoma (PA) is the most common pediatric brain tumor, accounting for ~20% of all brain tumors under the age of 20 [1–6]. They are slow growing WHO grade I tumors with an excellent prognosis reaching an overall survival rate up to 90% [7]. In some cases, the clinical course of the disease may be unpredictable with recurrence, progression or dissemination in 10–20% of cases [8–11].

Most PAs have alterations in the mitogen-activated protein kinase (MAPK) signalling pathway [5, 6, 12–15]. The most common genetic alteration found in PAs is the activating KIAA1549-BRAF fusion found in more than 60% of the cases [15–17]. Although less frequent, at least 8 additional partners (FAM131B, RNF130, CLCN6, MKRN1, GNA11, QK1, FZRI, and MACF1) have been found in BRAF fusions [18, 19]. All of them resulted in the loss of the regulatory domain with consequent activation of the MAPK pathway. Point mutations in BRAF have also been reported, including V600E that is found in 2–9% of PAs [16]. In addition to BRAF-related alterations, other mutations are reported, including FGFR1, NF1, KRAS as well as NTRK-family fusions [15, 16]. All these mutations are mutually exclusive and lead to the activation of the MAPK pathway, which is detected in nearly 100% of cases, thus making PA a single pathway disease [15, 16].

The identification of additional patient-specific mutations could provide crucial information regarding molecular pathways underlying non-responding PA and thus point to new therapeutic avenues. In this study, we identified a novel recurrent BRAF tandem insertion (p.V504_R506dup), which has an impact on MAPK phosphorylation and confers resistance to RAF inhibitors. We provided further evidence of the importance of this recurrent somatic mutation as a tumor-specific driver in PA development and its potential role as a guide for treatment strategies.

## Results

### Identification of a recurrent BRAF mutation

**Fig. 1:**
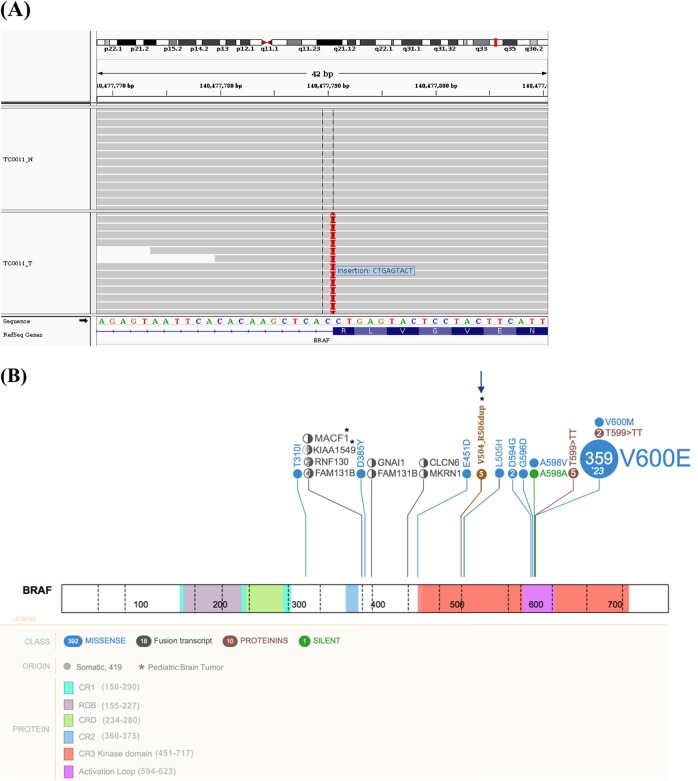
Identification of the BRAF p.V504_R506dup mutation in the PA patient TC0011. **a** Split-screen view from the integrative genomics viewer visualizing the 9 nucleotide insertion at the end of the exon 12, causing the repetition of the amino acid 504–506 VLR. **b** Schematic localization of the 9 bp insertion (p.V504_R506dup) in the Kinase domain at chr7:g.140477790 (indicated by an arrow). The middle panel shows BRAF functional domains and amino acid positions (1–766). The top panel depicts BRAF mutations reported in COSMIC (http://cancer.sanger.ac.uk/cosmic) and PeCan database (https://pecan.stjude.org) related to tumors in the central nervous system. The figure was built and adapted using the PeCan data portal (https://pecan.stjude.org/proteinpaint)

Whole-exome sequencing of the non-responding PA patient TC0011 led to the identification of three putative driver mutations in DTX1 (p.P331A), PAX3 (p.R270H) and BRAF (p.V504_R506dup). They were all confirmed by ultra-deep targeted re-sequencing. Only PAX3 (COSM6457105) was referenced in the COSMIC database. The BRAF p.V504_R506dup mutation, a heterozygous in-frame 9-bp tandem duplication (AGTACTCAG) at position chr7:g.140477790 results in the insertion of 3 additional amino acid residues at position 504 (Fig. 1). This mutation is clearly somatic as it was neither present in the corresponding germline nor in public database containing information about healthy individuals. We found 4 additional lower grade glioma cases (DO50162/TCGA-P5-A5EY, COSS2024527, GENIE-DFCI-009592, and ICGC_PA65 reported in Jones et al. [16]) carrying the same mutation in adult and pediatric public database (Table S1), demonstrating the recurrence of this BRAF mutation. This mutation was not observed in other tumor types, based on the available public databases, indicating specificity to pediatric PA. None of the known hotspot mutations in BRAF (V600E, L505H, and G466A), including the frequent KIAA1549-BRAF fusion as well as alterations in the *NF1*, *KRAS*, or *CRAF* genes, were found in these patients. As these mutations are usually mutually exclusive [16], it is tempting to propose that the recurrent BRAF p.V504_R506dup mutation might be a PA driver and diagnosis marker.

### Predicted impact of p.V504_R506dup on the BRAF structure

**Fig. 2:**
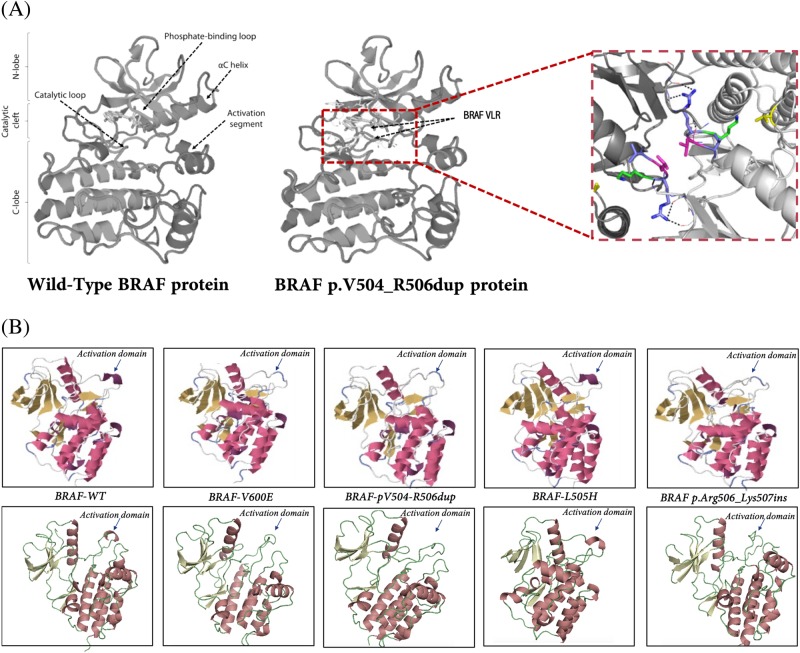
Predicted three-dimensional (3D) protein structure of the human BRAF and the impact of mutations on activation kinase domain. **a** The 3D protein structure of the wild type and mutated (p.V504_R506dup) BRAF proteins were modeled with PyMol and using the X-ray crystal structure from the protein data bank (PDB ID: 30G7). The impact of the p.V504_R506dup mutation is depicted by arrows. **b** Predicted molecular structures proposed by raptorX (top panel) and PyMol (bottom panel) for different alterations found in BRAF. The mutation p.V504_R506dup induces structure changes in the activation kinase domain that are comparable to BRAF V600E, BRAF L505H and the recently characterized p.Arg506_Lys507insLeuLeuArg [23–25]

Structural mapping showed that the BRAF p.V504_R506dup is located in the C-helix of the kinase domain N-terminal lobe (Fig. 2a) within the “RAF-selective” pocket determined by Leu505, Leu514, Phe516, and Phe596 and against the catalytic loop Lys507 and the 506 residue [20, 21], pointing inward toward a potential kinase catalytic cleft. Then we compared the predicted structures of p.V504_R506dup with 3 other BRAF mutations with known functional impact: V600E [22], L505H [23], and pArg506-Lys507ins [24]. Of note, the nearby residue 505, mutated (L505H) in melanoma and prostate cancer, has been associated with activation of MAPK signalling [23, 25]. The effect of the p.V504_R506dup on the molecular structure, especially on activating sites, appears to be similar to BRAF V600 according to X-Raptor and PyMol (Fig. 2b). Altogether, the structural model predicts that p.V504_R506dup is intrinsically resistant to BRAF inhibitors. Jones et al. suggested that p.V504_R506dup acts mainly as homodimer which could point to the non-effectiveness of RAF inhibitors on this mutation. Here, the computational modeling predict that p.V504_R506dup reduces the affinity of BRAF inhibitors for its drug target by changing the conformation of the αC-helix through promoting a productive on-state conformation of the AS (activation site) and the surrounding catalytic cleft.

### Impact of p.V504_R506dup on drug resistance

**Fig. 3:**
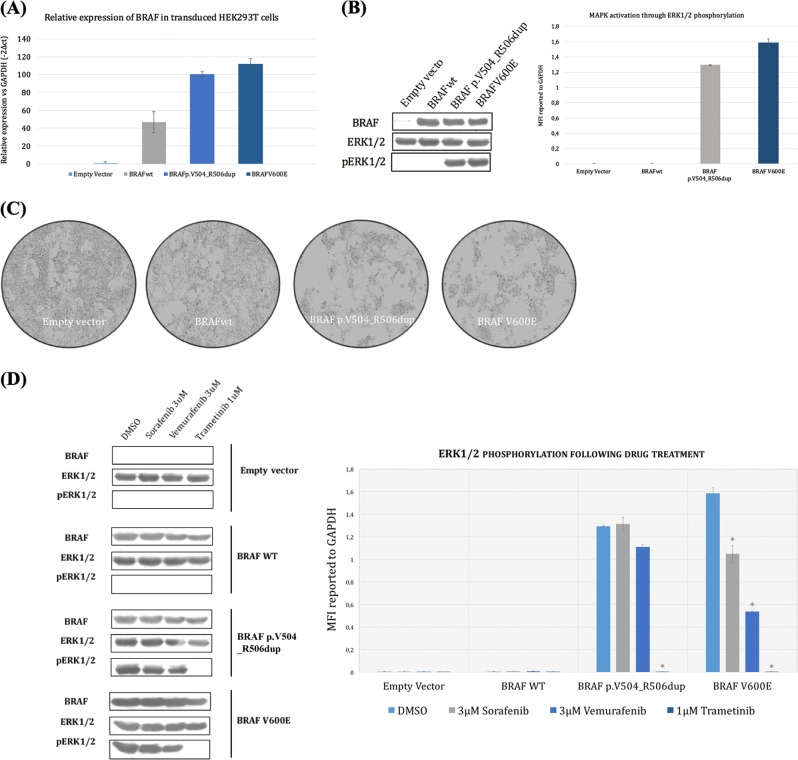
BRAF p.V504_R506dup mutation activates MAPK signaling and confers resistance to MEK inhibitors. **a** Relative expression of BRAF mutants (p.V504_R506dup and V600E) and wild type (WT) in transfected HEK293T cells. **b** Kinase activity of cells containing the WT or the mutants (p.V504_R506dup, V600) BRAF. The activity is measured by western blot (left) and Luminex phosphorylation measurements (right) using ERK1/2 as substrates. The total immunoprecipitated BRAF (BRAF), the phosphorylated level of ERK1/2 (pERK1/2) and the total level of ERK1/2 are shown. **c** Morphological examination of HEK293T cells 24 h after transfection with the empty vector or expressing BRAF-WT, BRAF p.V504_R506dup, or V600E. **d** BRAF p.V504_R506dup. Changes in pERK in HEK293T cells expressing the BRAF-WT and the mutants (p.V504_R506dup, V600E) after 18 h treatment with 3 µM of DMSO, 3 µM of vemurafenib, 3 µM of sorafenib or 1 µM of trametinib. Each sample was assayed in triplicate and error bars are used to indicate the standard deviations from the means. Similar results were obtained in two-independent transfections. * indicates a *P*-value <0.01 according to Student’s *t*-distribution

To assess the impact of p.V504_R506dup on the MAPK pathway, we transiently expressed the wild type or the mutant BRAF-derived constructs in HEK293T cells. These cells were selected because of their minimal baseline MAPK signaling and relatively low levels of phosphor-ERK1/2. A V600E construct was included as a positive control. The expression of the pV504_R506dup or V600E mutants resulted in increased levels of phospho-ERK1/2 but not its non-phosphorylated form (Fig. 3). In other words, the pV504_R506dup mutant displays similar activating and oncogenic potential as the prevalent V600E mutation, at least in HEK293T cells, as previously observed in Jones et al.. We then evaluated the impact of p.V504_R506dup in the response to RAF inhibitor (Vemurafenib and Sorafenib) and MEK inhibitor (Trametinib) by monitoring phosphorylation of the downstream signaling component of ERK1/2 in HEK293T cells. The WT BRAF did not confer resistance to any of the drugs tested (Fig. 3). Both p.V504_R506dup and V600E mutants were resistant to RAF inhibitors by persistent phosphor-ERK1/2 levels in the presence of increasing concentrations (0.1, 0.3, 1, 3 µM) of Sorafenib and Vermufinib. The expression level of CRAF was similar in all conditions tested (data not shown) suggesting that drug resistance was independent of dimerization with CRAF. Similarly to V600E, the mutant BRAF p.V504_R506dup had no effect on sensitivity to the MEK1/2 inhibitor (Fig. 3).

### Morphological changes of HEK293T cells transfected with the BRAF mutants

HEK293T cell expressing BRAF p.V504_R506dup or V600E exhibited a drastically elongated and refractile morphology with strongly reduced cell-substrate and cell–cell adhesion and crisscross growth (Fig. 3). In contrast, cells transfected with the empty vector or expressing BRAF-WT displayed representative fibroblast morphologies with the entire cell body attached to the substratum.

## Discussion

Here we reported a novel recurrent p.V504_R506dup BRAF mutation specific to pediatric Pilocytic Astrocytoma. One of these patients (TC0011) was a young adult with recurrence and metastasis in the central nervous system illustrating the need to further improve our knowledge of poorer clinical outcomes. A combination of structural mapping and in vitro cell assay indicated that p.V504_R506dup confers resistance to BRAF inhibitors (Sorafenib, vemurafenib) and sensitivity to MEK1/2 inhibitors (trametinib).

We showed that the p.V504_R506dup has a predicted effect on the conformation of the αC-helix through the addition of hydrophobic bonds in the regions where the adenine ring of ATP binds. This could reduce the affinity of BRAF inhibitors. Our results are comparable to studies on other nearby mutants, including p.Arg506_Lys507insLeuLeuArg [24] and the R509 deletion [26]. A different resistance mechanisms has been described for L505, a substitution found in melanomas [23, 25] for which the substitution affects the binding of Vemurafenib probably due to the presence of the larger polar amino acid distorting the peptide backbone of the activation segment at the contact site. The occurrence of alterations affecting the residues 505–509 highlights the importance of this region in MAPK activation and could explain resistant phenotype to RAF inhibitors observed in HEK293T cell lines. Further investigations in stable modified cell lines could provide valuable insights into the mechanisms underlying the phenotype observed.

We have shown that p.V504_R506dup mutant functions as homodimer, does not appear to form strong heterodimers, and that drug resistance is independent of dimerization with CRAF. This is important in the context that most of the existing RAF inhibitors can paradoxically activate the RAF kinase in tumors wild type for BRAF through induced conformational changes and heterodimerization that triggers RAS-dependent transactivation and leads to MEK/ERK phosphorylation [27–32].

## Conclusion

The knowledge about molecular genetics behind development of PA has increased tremendously in recent years. The recurrent BRAF p.V504_R506dup reported in this paper further emphasizes the central role of *BRAF* in PA tumorigenesis. The occurrence of an increasing number of *BRAF* alterations and possibility for MAPK pathway targeted therapy highlights the importance of robust methods for fast and cost-effective detection of *BRAF* deregulations to guide diagnosis, prognosis, and accurately targeted therapy.

## Materials and methods

### Patients’ description

The patient TC0011 was diagnosed with a pilocytic astrocytoma of the optic pathways at 6 years and 7 months of age in the Division of Hematology-Oncology at the Sainte-Justine Hospital (Montreal, Canada). The patient had no clinical history of neurofibromatosis type 1. At diagnosis, he received chemotherapy with carboplatin and vincristine (Pediatric Oncology Group (POG) protocol A9952) followed by a total of 20 sessions of 1.8 Gy radiotherapy. Four years and 6 months after the end of treatment, the tumor progression was detected by imaging and a concomitant visual deterioration. He then started another cycle of chemotherapy (out of study, POG A9952). After 1 year of treatment, the patient then 14 years old achieved partial remission. The residual vision was slight but stable and the patient was functional. At 18 years, the patient presented with increased fatigability and tremors in the lower right limb with foot drop. No headache or visual deterioration was noted. MRI scan revealed a very heterogeneous astrocytoma with partly solid and partly cystic lesions. The MRI showed a slight progression of a cyst located in the posterior part of the lesion facing the left cerebral peduncle. Radiotherapy and surgery options were excluded. Chemotherapy, including decadron was urgently started using the COG ACNS1022 clinical trial protocol. Few months later, the MRI scan revealed a marked progression of the solid and cystic components of the lesion with concomitant neurological deterioration. Then the patient underwent surgery (stereotactic biopsy from the left hole and installation of a catheter in the tumoral cystic portion). He was then invited to participate to our institutional precision medicine program in pediatric cancer called TRICEPS. This study was approved by our Research Ethics Board (REB) and consent forms were obtained. An informed consent form approved by the local IRB/REB and updated annually was provided for the patients.

The querying of four public database, TCGA (The Cancer Genome Atlas; *cancergenome.nih.gov)*, TARGET (Therapeutically Applicable Research To Generate Effective Treatments; *ocg.cancer.gov/programs/target*), ICGC (International Cancer Genome consortium; https://icgc.org) and GENIE-AACR (https://www.aacr.org/Research/Research/Pages/aacr-project-genie.aspx) led to the identification of four additional lower grade glioma patients carrying the BRAF p.V504_R506dup alteration same mutation (Table S1).

### Whole-exome sequencing and bioinformatics analysis

DNA was extracted from a peripheral blood sample (normal material) and brain biopsy (relapse material) using standard protocols [33]. Whole exomes were captured in solution using the SureSelect XT Clinical Research Exome kit (Agilent genomics) as per the manufacturer’s instructions and sequenced on the Illumina HiSeq 2500 system (Paired-end, 2 × 100 bp; mean coverage on targeted region of 293X for relapse and 191X for germline) at the Integrated Clinical Genomics Centre In Pediatrics (CHU Sainte-Justine). Bioinformatic analysis was performed as described elsewhere [34]. Briefly, alignment of the resulting exome reads to the hg19 reference genome was performed using BWA (version 0.7.7) [35]. PCR duplicates were removed using Picard (http://broadinstitutegithubio/picard/). Genotype quality score recalibration was performed using the Genome Analysis ToolKit (GATK Version 3.3) [36]. Somatic single nucleotide variants (SNVs) and small indels were called from pileup files using Varscan [37]. The sequencing information from the corresponding germline genome was use to confirm the somatic status of the mutations. The tumor-specific SNVs and indels were confirmed by MiSeq target sequencing (>×1000 coverage) at the McGill University and Génome Québec Innovation Center.

### Identification of putative cancer driver mutations

ANNOVAR (v2015Jun17) [38] and Oncotator (v1.5.3.0) [39] were used to annotate somatic splice site variants, non-synonymous SNVs and frameshift small indels (Table S3). Variants were queried against publicly available datasets such as 1000 Genomes (Nature 2012) and NHLBI GO Exome Sequencing Project (ESP) (http://evs.gs.washington.edu/EVS/URL) to filter out SNVs with minor allele frequency >0.01. The predicted functional impact of non-synonymous variants and small indels was assessed using Sift (v1.03) [40], Polyphen2 (v2.2.2) [41], MutationTaster 2 [42] as previously described [34]. Further annotation of the remaining SNVs was done using public database publishing correlations of drugs/variant sensitivity profiles or clinical trials (e.g., PharmGKB; dgidb (http://dgidb.genome.wustl.edu/); FDA (http://www.fda.gov/drugs/); Health Canada (http://www.hc-sc.gc.ca/), etc).

### Cloning and expression constructs

The BRAF-derived constructs containing the mutations p.V504_R506dup or V600E were generated by Site-directed mutagenesis (Kit quickchange XL II -Agilent) using the BRAF wild-type plasmid (HsCD00379096, DF/HCC DNA Resource Core at Harvard Medical School) and the oligonucleotides listed in Table S2. The resulting PCR fragments were subcloned into a pENTR3C gateway vector (Invitrogen). The integrity of the resulting mutant constructs was validated by Sanger sequencing (McGill University and Génome Québec Innovation Centre) before being transferred into the gateway pCDNA3.2 vector.

### Cell culture and drug treatment

Human embryonic kidney 293T cells (HEK293T; ATCC CRL-3216) were cultured in Dulbecco’s modified Eagle’s medium (DMEM) (Wisent) supplemented with 10% heat-inactivated FBS. Twenty-four hours before transfection, cells were plated at a density of 3 × 10^5^ cells per well in 6-well plates. The cells were then transfected with 200 ng of BRAF-derived constructs (p.V504_R506dup, V600E or WT) or an empty-pCDNA3.2 as a control using lipofectamin^TM^ 2000 reagent (Thermo Fisher Scientific) in accordance with the manufacturer’s protocol. For drug treatment, the transfected cells (24 h post transfection) were treated for 18 h with either Vemurafenib (Ss-364634; Lot # F2515), Sorafenib (sc-220125; Lot # F0215), Trametinib (sc-364639; Lot # F1715) at various concentration or DMSO as control.

### RNA extraction and RT-qPCR

Total RNA was extracted from transduced HEK293T cells using RNeasy mini Kit (Qiagen), reverse-transcribed into cDNA using the QuantiTect Reverse Transcription Kit (Qiagen), and quantified by qPCR amplifications (triplicates) on the ABI Prism 7000 Sequence Detection System (Thermo Fisher Scientific) using SYBR Green PCR Master Mix (Applied Biosystems). The cycling parameters were 95 °C for 10 min, 40 cycles (95 °C for 15 s, 60 °C for 1 min) followed by a denaturation curve at 60 °C. GAPDH was used as reference gene. Expression values were calculated as 2^−(ΔΔCT)^, as per Livak and Schmittgen (Livak et al., 2001) and reported to GAPDH expression. The vertical bars show 95% confidence intervals according to Student’s *t*-distribution. Primers sequences used are listed in Table S2.

### Protein isolation and western blot analysis

Proteins were isolated with protein lysis buffer obtained from the Panel 9-plex (Milliplex Map lysis Buffer #43-040). Protein concentrations were quantified by BCA assay (Pierce, Rockford, IL, USA) and protein (150 μg) was loaded on Bio-Rad Mini-Protean gels (TGX pre-cast anyKD) and transferred onto PVDF western blotting membranes (Roche Diagnostics, Indianapolis, IN, USA). Membranes were blocked with 5% non-fat dry milk in TBST (TBS, 0.05% Tween 20) and probed with 1:200 anti-phospho ERK1/2 (Cell Signaling, phospho-p44/42 MAPK pThr202/Tyr204- # 4370, Danvers, MA, USA), 1:200 anti- ERK1/2 (Cell Signaling, p44/42 MAPK-ERK1/2-Cell Signaling, #4695, Danvers, MA, USA), 1:500 anti-BRAF (Santa Cruz Biotech, # sc-166), or 1:10 000 anti-GAPDH (Ambion by Life technologies, #AM4300) in 5% non-fat dry milk in TBST (TBS, 0.05% Tween 20), followed by anti-mouse for GAPDH or anti-rabbit for others, IgG-HRP:Sc358914 and IgG-HRP: Sc-20177-labeled secondary antibody (Santa Cruz Biotech), respectively.

### Luminex analysis

Protein phosphorylation was determined using the Luminex 2000, a flow cytometry-based system, combined with 9-plex Multi-Pathway Magnetic Bead Panel (Millipore #46-680MAG, Amsterdam, Netherlands) following the manufacturer’s protocol. Cell lysates (Millipore #47-210) was used as a negative control and GAPDH beads (Millipore #46-667MAG) were added to correct for protein loading.

### Structural modeling

The experimental determined structures of human BRAF were downloaded from the Protein Data Bank (http://www.rcsb.org) using the entries 3C4C | 30G7 or 1UWJ [43–45]. The structural modeling of BRAF bound to drugs and the structural elucidation of mutants were generated using molecular visualization PyMol (Educational version, Delano Inc, CA, USA; *pymol.org*) and RaptorX [46] softwares.

## Supplementary information

Table S1.A

Table S1.B

Table S2

Table S3

Supp_Data_For_reviewer

Supp_Data_For_reviewer

Supp_Data_For_reviewer

## References

[1] Louis DN, Ohgaki H, Wiestler OD, Cavenee WK, Burger PC, Jouvet A (2007). The 2007 WHO classification of tumours of the central nervous system. Acta Neuropathol.

[2] Potter N, Karakoula A, Phipps KP, Harkness W, Hayward R, Thompson DN (2008). Genomic deletions correlate with underexpression of novel candidate genes at six loci in pediatric pilocytic astrocytoma. Neoplasia.

[3] Pfister S, Janzarik WG, Remke M, Ernst A, Werft W, Becker N (2008). BRAF gene duplication constitutes a mechanism of MAPK pathway activation in low-grade astrocytomas. J Clin Investig.

[4] Jones DT, Ichimura K, Liu L, Pearson DM, Plant K, Collins VP (2006). Genomic analysis of pilocytic astrocytomas at 0.97 Mb resolution shows an increasing tendency toward chromosomal copy number change with age. J Neuropathol Exp Neurol.

[5] Belirgen M, Berrak SG, Ozdag H, Bozkurt SU, Eksioglu-Demiralp E, Ozek MM (2012). Biologic tumor behavior in pilocytic astrocytomas. Childs Nerv Syst.

[6] Sanoudou D, Tingby O, Ferguson-Smith MA, Collins VP, Coleman N (2000). Analysis of pilocytic astrocytoma by comparative genomic hybridization. Br J Cancer.

[7] Budka H (1975). Partially resected and irradiated cerebellar astrocytoma of childhood: malignant evolution after 28 years. Acta Neurochir.

[8] Krieger MD, Gonzalez-Gomez I, Levy ML, McComb JG (1997). Recurrence patterns and anaplastic change in a long-term study of pilocytic astrocytomas. Pediatr Neurosurg.

[9] Stuer C, Vilz B, Majores M, Becker A, Schramm J, Simon M (2007). Frequent recurrence and progression in pilocytic astrocytoma in adults. Cancer.

[10] Katsetos CD, Krishna L (1994). Lobar pilocytic astrocytomas of the cerebral hemispheres: I. Diagnosis and nosology. Clin Neuropathol.

[11] Rodriguez FJ, Scheithauer BW, Burger PC, Jenkins S, Giannini C (2010). Anaplasia in pilocytic astrocytoma predicts aggressive behavior. Am J Surg Pathol.

[12] Gajjar A, Pfister SM, Taylor MD, Gilbertson RJ (2014). Molecular insights into pediatric brain tumors have the potential to transform therapy. Clin Cancer Res.

[13] Orr LC, Fleitz J, McGavran L, Wyatt-Ashmead J, Handler M, Foreman NK (2002). Cytogenetics in pediatric low-grade astrocytomas. Med Pediatr Oncol.

[14] Jones DT, Gronych J, Lichter P, Witt O, Pfister SM (2012). MAPK pathway activation in pilocytic astrocytoma. Cell Mol Life Sci.

[15] Zhang J, Wu G, Miller CP, Tatevossian RG, Dalton JD, Tang B (2013). Whole-genome sequencing identifies genetic alterations in pediatric low-grade gliomas. Nat Genet.

[16] Jones DT, Hutter B, Jager N, Korshunov A, Kool M, Warnatz HJ (2013). Recurrent somatic alterations of FGFR1 and NTRK2 in pilocytic astrocytoma. Nat Genet.

[17] Bergthold G, Bandopadhayay P, Bi WL, Ramkissoon L, Stiles C, Segal RA (2014). Pediatric low-grade gliomas: how modern biology reshapes the clinical field. Biochim Biophys Acta.

[18] Ross JS, Wang K, Chmielecki J, Gay L, Johnson A, Chudnovsky J (2016). The distribution of BRAF gene fusions in solid tumors and response to targeted therapy. Int J Cancer.

[19] Collins VP, Jones DT, Giannini C (2015). Pilocytic astrocytoma: pathology, molecular mechanisms and markers. Acta Neuropathol.

[20] Karoulia Z, Wu Y, Ahmed TA, Xin Q, Bollard J, Krepler C (2016). An integrated model of RAF inhibitor action predicts inhibitor activity against oncogenic BRAF signaling. Cancer Cell.

[21] Haling JR, Sudhamsu J, Yen I, Sideris S, Sandoval W, Phung W (2014). Structure of the BRAF-MEK complex reveals a kinase activity independent role for BRAF in MAPK signaling. Cancer Cell.

[22] Bollag G, Hirth P, Tsai J, Zhang J, Ibrahim PN, Cho H (2010). Clinical efficacy of a RAF inhibitor needs broad target blockade in BRAF-mutant melanoma. Nature.

[23] Choi J, Landrette SF, Wang T, Evans P, Bacchiocchi A, Bjornson R (2014). Identification of PLX4032-resistance mechanisms and implications for novel RAF inhibitors. Pigment Cell Melanoma Res.

[24] Heritier S, Helias-Rodzewicz Z, Chakraborty R, Sengal AG, Bellanne-Chantelot C, Thomas C (2017). New somatic BRAF splicing mutation in Langerhans cell histiocytosis. Mol Cancer.

[25] Wagenaar TR, Ma L, Roscoe B, Park SM, Bolon DN, Green MR (2014). Resistance to vemurafenib resulting from a novel mutation in the BRAFV600E kinase domain. Pigment Cell Melanoma Res.

[26] Chen SH, Zhang Y, Van Horn RD, Yin T, Buchanan S, Yadav V (2016). Oncogenic BRAF deletions that function as homodimers and are sensitive to inhibition by RAF dimer inhibitor LY3009120. Cancer Discov.

[27] Heidorn SJ, Milagre C, Whittaker S, Nourry A, Niculescu-Duvas I, Dhomen N (2010). Kinase-dead BRAF and oncogenic RAS cooperate to drive tumor progression through CRAF. Cell.

[28] Garnett MJ, Marais R (2004). Guilty as charged: B-RAF is a human oncogene. Cancer Cell.

[29] Kandoth C, McLellan MD, Vandin F, Ye K, Niu B, Lu C (2013). Mutational landscape and significance across 12 major cancer types. Nature.

[30] Poulikakos PI, Persaud Y, Janakiraman M, Kong X, Ng C, Moriceau G (2011). RAF inhibitor resistance is mediated by dimerization of aberrantly spliced BRAF(V600E). Nature.

[31] Hatzivassiliou G, Song K, Yen I, Brandhuber BJ, Anderson DJ, Alvarado R (2010). RAF inhibitors prime wild-type RAF to activate the MAPK pathway and enhance growth. Nature.

[32] Freeman AK, Ritt DA, Morrison DK (2013). Effects of Raf dimerization and its inhibition on normal and disease-associated Raf signaling. Mol Cell.

[33] Baccichet A, Qualman SK, Sinnett D (1997). Allelic loss in childhood acute lymphoblastic leukemia. Leuk Res.

[34] Spinella JF, Cassart P, Richer C, Saillour V, Ouimet M, Langlois S (2016). Genomic characterization of pediatric T-cell acute lymphoblastic leukemia reveals novel recurrent driver mutations. Oncotarget.

[35] Li H, Durbin R (2010). Fast and accurate long-read alignment with Burrows-Wheeler transform. Bioinformatics.

[36] McKenna A, Hanna M, Banks E, Sivachenko A, Cibulskis K, Kernytsky A (2010). The Genome Analysis Toolkit: a MapReduce framework for analyzing next-generation DNA sequencing data. Genome Res.

[37] Koboldt DC, Chen K, Wylie T, Larson DE, McLellan MD, Mardis ER (2009). VarScan: variant detection in massively parallel sequencing of individual and pooled samples. Bioinformatics.

[38] Wang K, Li M, Hakonarson H (2010). ANNOVAR: functional annotation of genetic variants from high-throughput sequencing data. Nucleic Acids Res.

[39] Ramos AH, Lichtenstein L, Gupta M, Lawrence MS, Pugh TJ, Saksena G (2015). Oncotator: cancer variant annotation tool. Hum Mutat.

[40] Kumar P, Henikoff S, Ng PC (2009). Predicting the effects of coding non-synonymous variants on protein function using the SIFT algorithm. Nat Protoc.

[41] Adzhubei IA, Schmidt S, Peshkin L, Ramensky VE, Gerasimova A, Bork P (2010). A method and server for predicting damaging missense mutations. Nat Methods.

[42] Schwarz JM, Cooper DN, Schuelke M, Seelow D (2014). MutationTaster2: mutation prediction for the deep-sequencing age. Nat Methods.

[43] Tsai J, Lee JT, Wang W, Zhang J, Cho H, Mamo S, et al. Discovery of a selective inhibitor of oncogenic B-Raf kinase with potent antimelanoma activity. Proc Natl Acad Sci USA. 2008;105:3041–6. 10.1073/pnas.0711741105 PMC2268581 18287029

[44] Rahman MA, Salajegheh A, Smith RA, Lam AK (2014). BRAF inhibitors: from the laboratory to clinical trials. Crit Rev Oncol/Hematol.

[45] Roskoski R (2016). Classification of small molecule protein kinase inhibitors based upon the structures of their drug-enzyme complexes. Pharmacol Res.

[46] Ma J, Wang S, Zhao F, Xu J (2013). Protein threading using context-specific alignment potential. Bioinformatics.

