# Supplementary material for: Recurrent somatic BRAF insertion (p.V504_R506dup): a tumor marker and a potential therapeutic target in pilocytic astrocytoma
Source: Oncogene. 2018 Dec 21;38(16):2994–3002. doi: 10.1038/s41388-018-0623-3 (PMC6484687; doi:10.1038/s41388-018-0623-3)
Supplement: Supplementary file 1 — Table S1.A [file 41388_2018_623_MOESM1_ESM.pdf]

TABLE S1A. Clinical data of patients harboring BRAF p.V504_R506dup, including those identified within public databases : TCGA, TARGET, GENIE-AACR and ICGC

Patient #	Patient Identification	Tumor	Age at diagnosis	Sex	status after Treatment	Current Statut	Source
P1	TC0011	Pilocytic Astrocytomas	6	male	relapse	alive	CHU Ste-Justine
P2	DO50162/TCGA-P5-A5EY	Astrocytomas diffuse	21	male	NA	alive	TCGA-US
P3	COSS2024527	Lower Grade Glioma (PA)	14	male	NA	Unknown	COSMIC
P4	ICGC_PA65	Pilocytic Astrocytomas	16	male	NA	alive	COSMIC/Jones et al., 2013
P5	GENIE-DFCI-009592	Pilocytic Astrocytomas	<18	male	NA	Unknown	GENIE-AACR
