# Supplementary material for: Recurrent somatic BRAF insertion (p.V504_R506dup): a tumor marker and a potential therapeutic target in pilocytic astrocytoma
Source: Oncogene. 2018 Dec 21;38(16):2994–3002. doi: 10.1038/s41388-018-0623-3 (PMC6484687; doi:10.1038/s41388-018-0623-3)
Supplement: Supplementary file 2 — Table S1.B [file 41388_2018_623_MOESM2_ESM.pdf]

| p | Nb of cases | Description |
|---|---|---|
| **TCGA-LGG-US** | 516 | Lower Grade Glioma cases examined |
| | 276 | Brain Glioblastoma Multiforme-TCGA, US |
| | 499 | Pediatric Brain Cancer - DE |
| **ICGC** | 283 | Brain Lower Grade Glioma - TCGA, US |
| | 186 | Pediatric Brain Tumor - Multiple subtypes |
| | 112 | Pediatric Medulloblastoma - CA |
| | 28 | Pilocytic Astrocytoma |
| | 212 | Astrocytoma |
| | 215 | Anaplastic Astrocytoma |
| **GENIE AACR** | 379 | Glioblastoma |
| | 799 | Glioblastoma Multiforme |
| | 33 | Low-Grade Glioma, NOS |
| **TARGET** | 222 | Neuroblastome |
