# Supplementary material for: Recurrent somatic BRAF insertion (p.V504_R506dup): a tumor marker and a potential therapeutic target in pilocytic astrocytoma
Source: Oncogene. 2018 Dec 21;38(16):2994–3002. doi: 10.1038/s41388-018-0623-3 (PMC6484687; doi:10.1038/s41388-018-0623-3)
Supplement: Supplementary file 3 — Table S2 [file 41388_2018_623_MOESM3_ESM.pdf]

Table S2. Primers used for mutagenesis, RT-PCR and RT-qPCR

Primer name	Sequence 5'-3'	Used for
BRAF_INS9nt_after_1517-as	AAAAATGAAGTAGGAGTACTCAGAGTACTCAGGAAAACACGACATGTGAATATCC	primer used for construction of the 9 nucleotide duplication in pENTR3C and in pCDNA3.2 vector
BRAF_ins9nt_after_1517-as	GGATATTCACATGTCGTGTTTTCCTGAGTACTCTGAGTACTCCTACTTCATTTTT	
BRAF_V600E-Fw	CCACTCCATCGAGAT TTCTCTGTAGCTAGACCAAAAT	primer used for construction of the V600E mutation in pENTR3C and pCDNA3.2 vector
BRAF_V600E-Fw	ATTTTGGTCTAGCTACAGAGAAATCTCGATGGAGTG	
BRAF-Exon 11-Fw	ACACTTGGTAGACGGGAC TC	primer used or detection of the 9 nucleotide duplication in patient tumoral material and in cell lines by RT-PCR
BRAF-Exon 13-Rv	GGAGCCCTCACACCACT	
GADPH expression- Fw	TGCACCACCAACTGCTTAGC	Primers used for RT-qPCR of GAPDH
GADPH expression -Rv	GGCATGGACTGTGGTCATGAG	
BRAF- Exon12-FW	gcacctacacctcagcagtt	primer used for Sanger Sequencing of V600 and INS constructs. Primers for total cDNA sequencing available upon request.
BRAF-Exon-14-RV	gtggatgattgacttggcgtg	
KIA1549 - BRAF- Fw	GAGGGACGCAGGAGATAAGA	primer used to check the presence of the reccurent fusion KIAA1549-BRAF
KIA1549 - BRAF - RV	CCAGGAAGAGCTCACGGATA	
