# Supplementary material for: Recurrent somatic BRAF insertion (p.V504_R506dup): a tumor marker and a potential therapeutic target in pilocytic astrocytoma
Source: Oncogene. 2018 Dec 21;38(16):2994–3002. doi: 10.1038/s41388-018-0623-3 (PMC6484687; doi:10.1038/s41388-018-0623-3)
Supplement: Supplementary file 4 — Table S3 [file 41388_2018_623_MOESM4_ESM.pdf]

Table S3. **Somatic mutation found by WES of tumoral material of the TC0011. Mutations were validated by Sanger and ultra-deep sequencing**

Chr	Start	End	Ref	Alt	DP	Func.refgene	Gene.refgene	ExonicFunc.	SOMATIC
chr12	113531016	113531016	C	G	353	exonic	DTX1	nonsynonyn	yes
chr2	223086090	223086090	C	T	308	exonic	PAX3	nonsynonyn	yes
chr7	140477790	140477790	-	CTGAGTACT	52	splicing	BRAF(NM_004333:e	NA	yes
