# Supplementary material for: Recurrent somatic BRAF insertion (p.V504_R506dup): a tumor marker and a potential therapeutic target in pilocytic astrocytoma
Source: Oncogene. 2018 Dec 21;38(16):2994–3002. doi: 10.1038/s41388-018-0623-3 (PMC6484687; doi:10.1038/s41388-018-0623-3)

GEL BRAF / ERK-PHOSPHO (WT and empty vector) – Figure 3


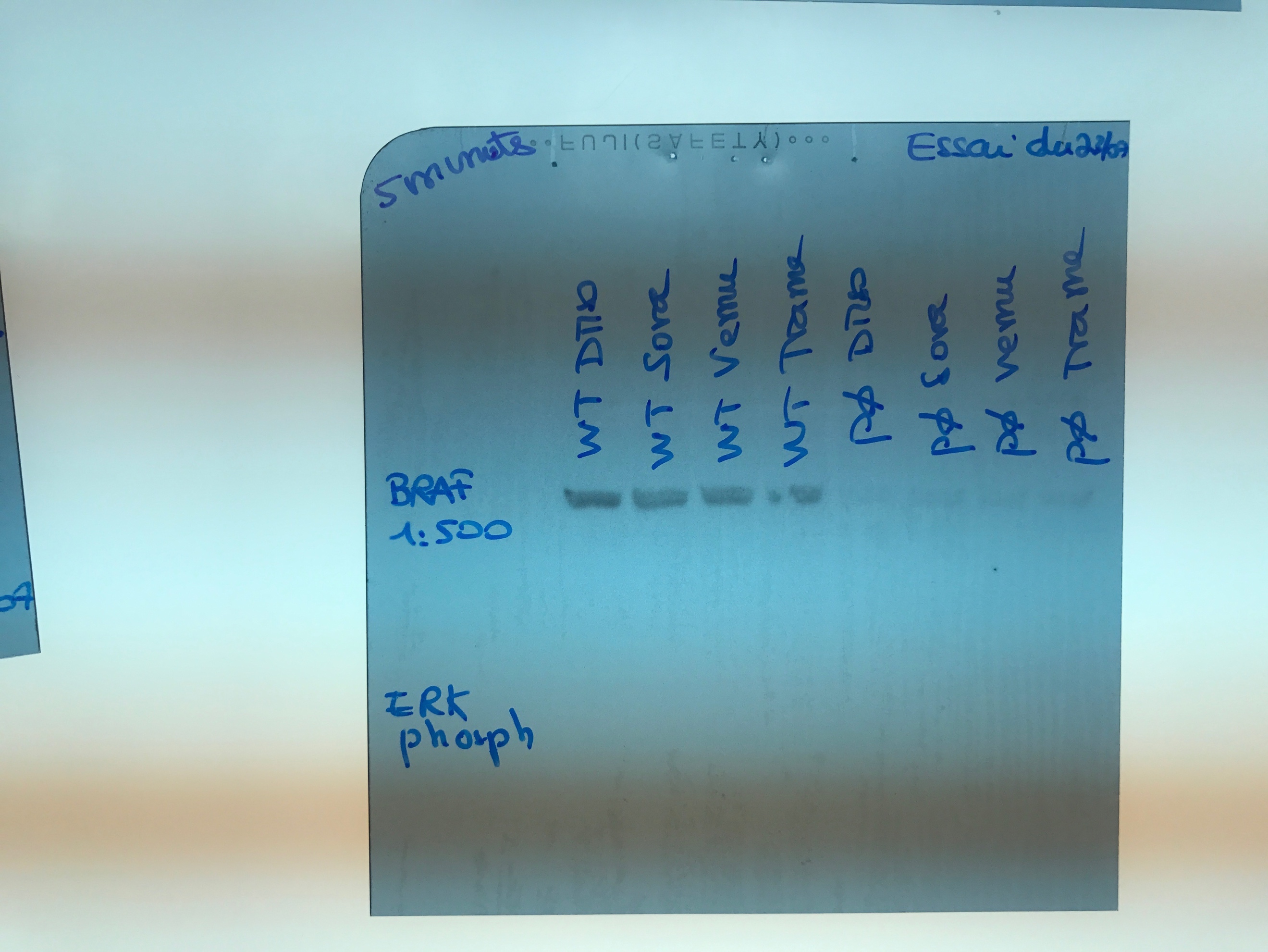


GEL BRAF / ERK-PHOSPHO (p.V504_506dup) – Figure 3


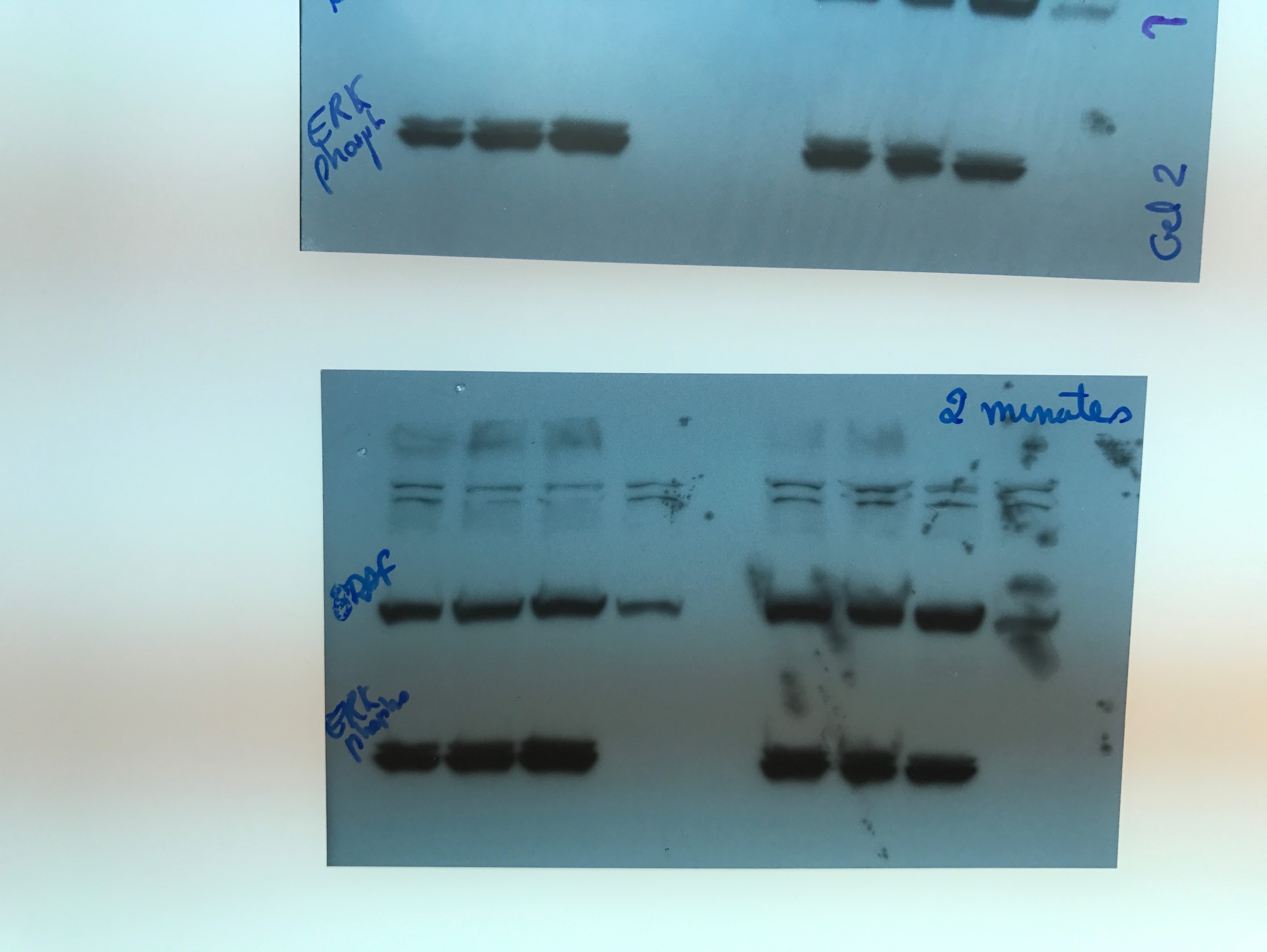


GEL BRAF / ERK-PHOSPHO (V600) - Figure 3


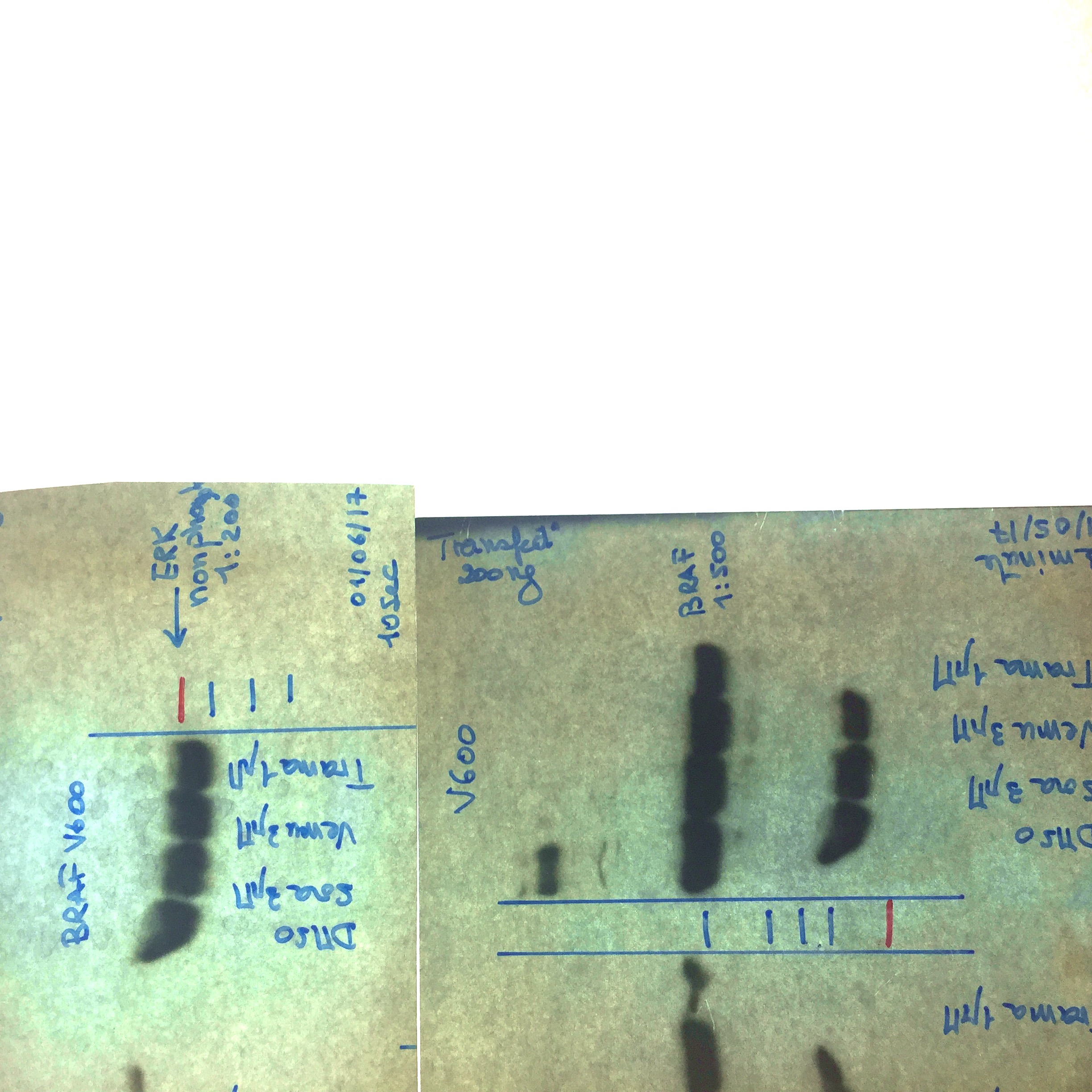


GEL ERK-NONPHOSPHO (WT and empty vector) Figure 3


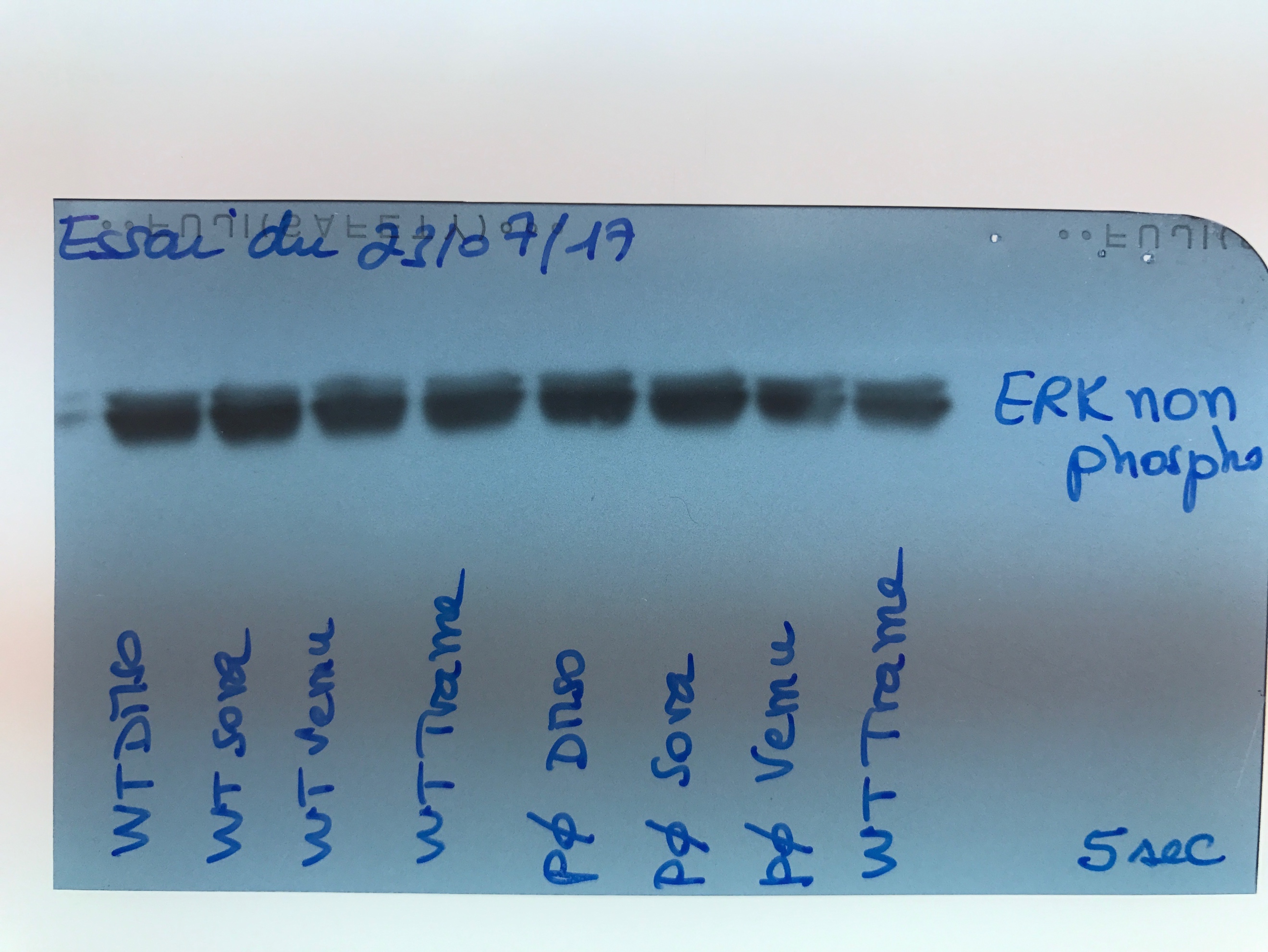


GEL ERK-NONPHOSPHO (Ins) Figure 3


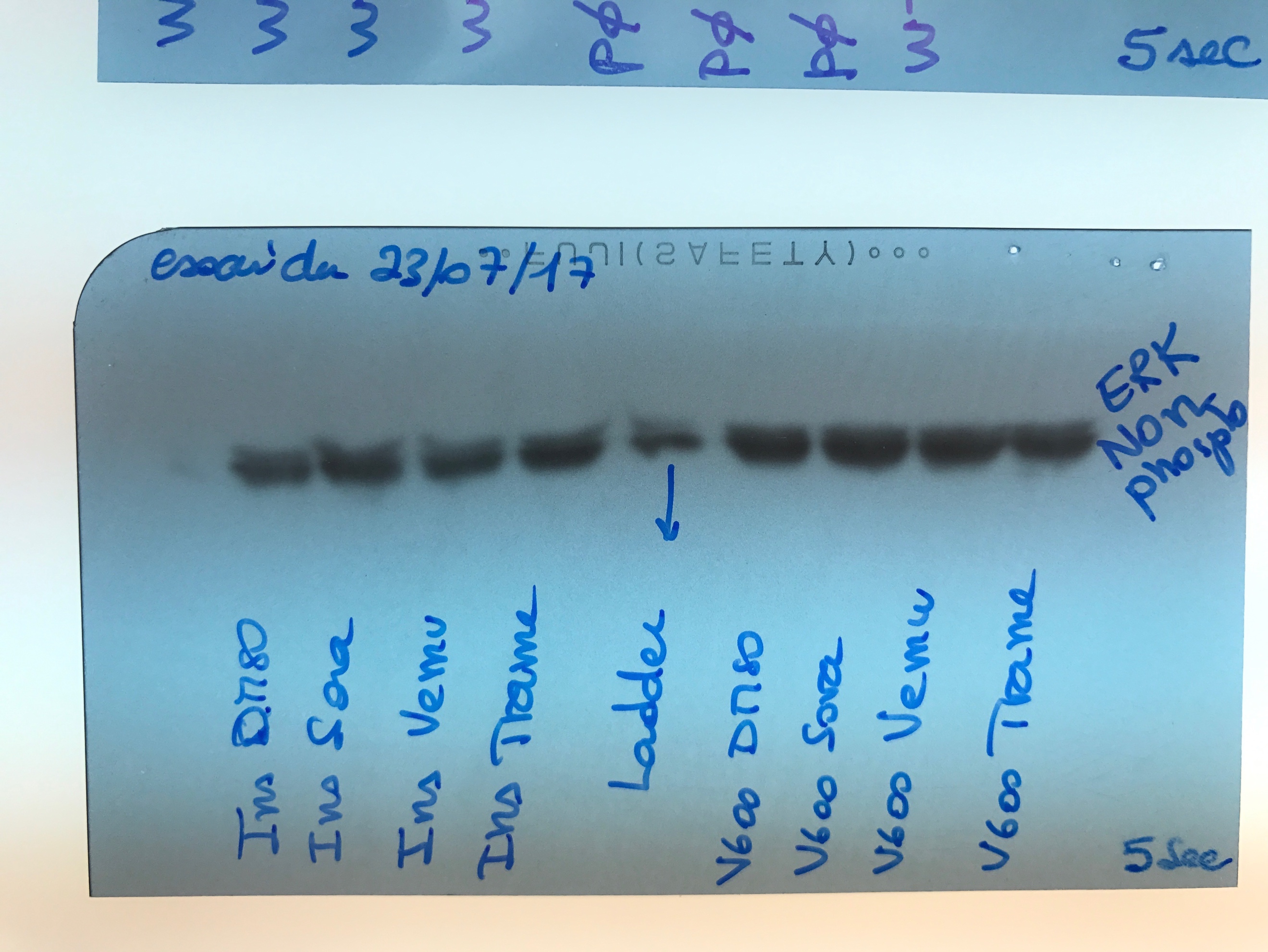


Gel ERK- NONPHOSPHO (V600E) Figure 3


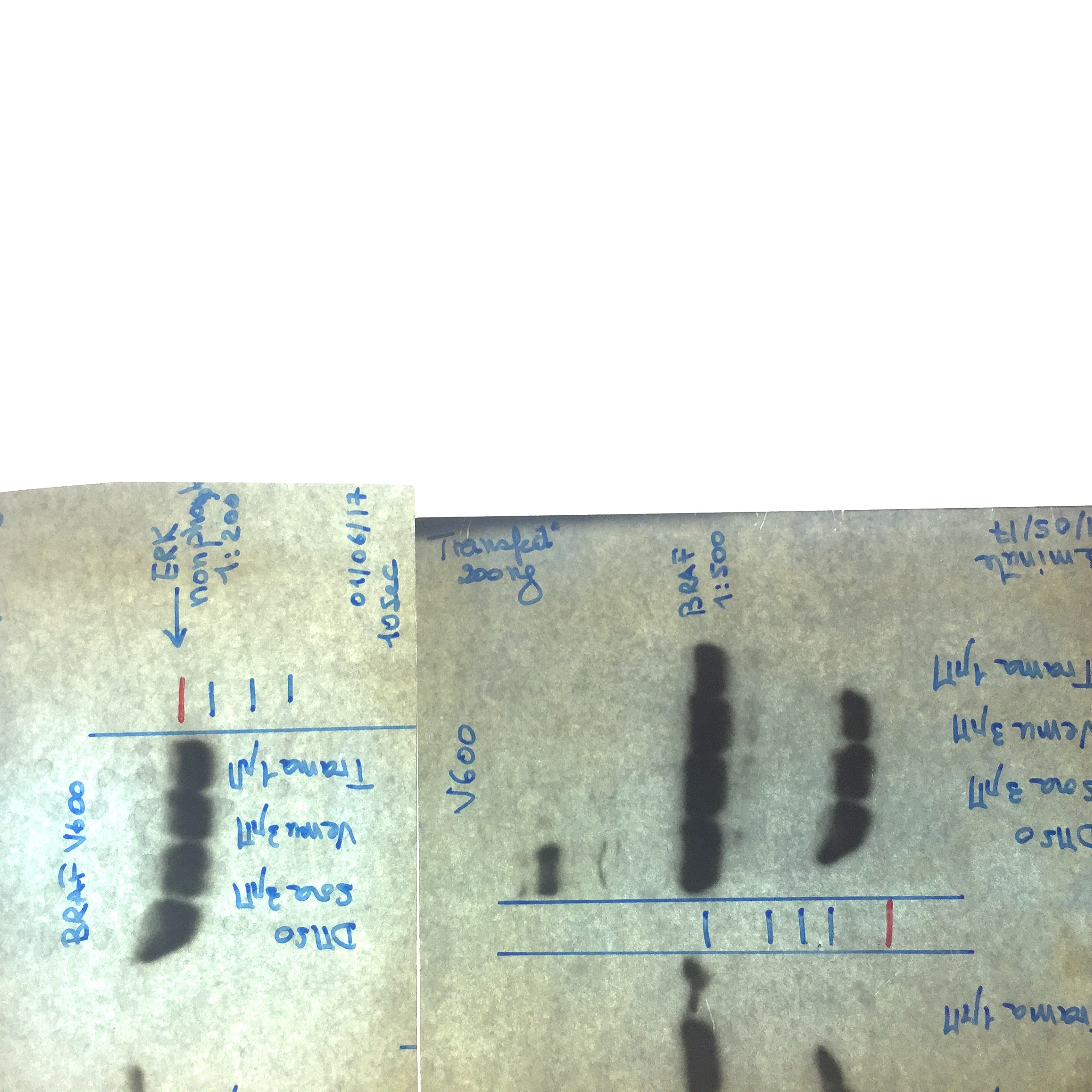

Supplement: Supplementary file 5 — Supp_Data_For_reviewer [file 41388_2018_623_MOESM5_ESM.docx]
