# Supplementary material for: Recurrent somatic BRAF insertion (p.V504_R506dup): a tumor marker and a potential therapeutic target in pilocytic astrocytoma
Source: Oncogene. 2018 Dec 21;38(16):2994–3002. doi: 10.1038/s41388-018-0623-3 (PMC6484687; doi:10.1038/s41388-018-0623-3)

Proliferation over 8 days

- There is no significant difference between the cell lines when seeded at low confluence.

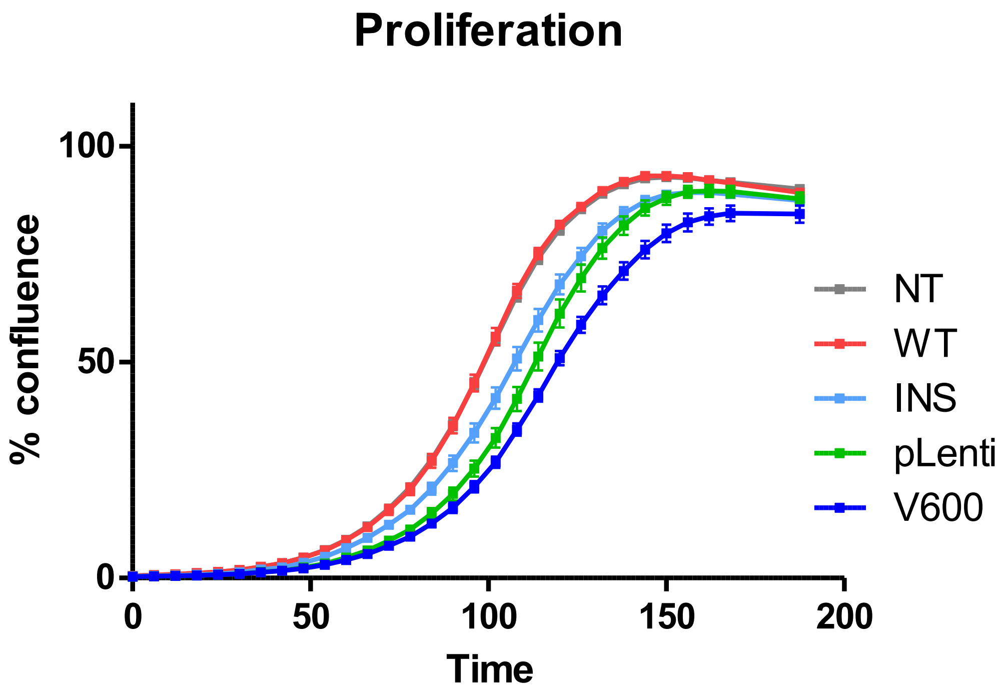

Proliferation at high density over 4 days

- When cells are seeded at higher density (about 50% confluence), the mutant V600E and the p.V504_R506dup show a distinct comportement. The two mutants don't reach full confluence and their decay is faster than the other cell lines.
- This difference is significative. (One-Way ANOVA + tuckey)

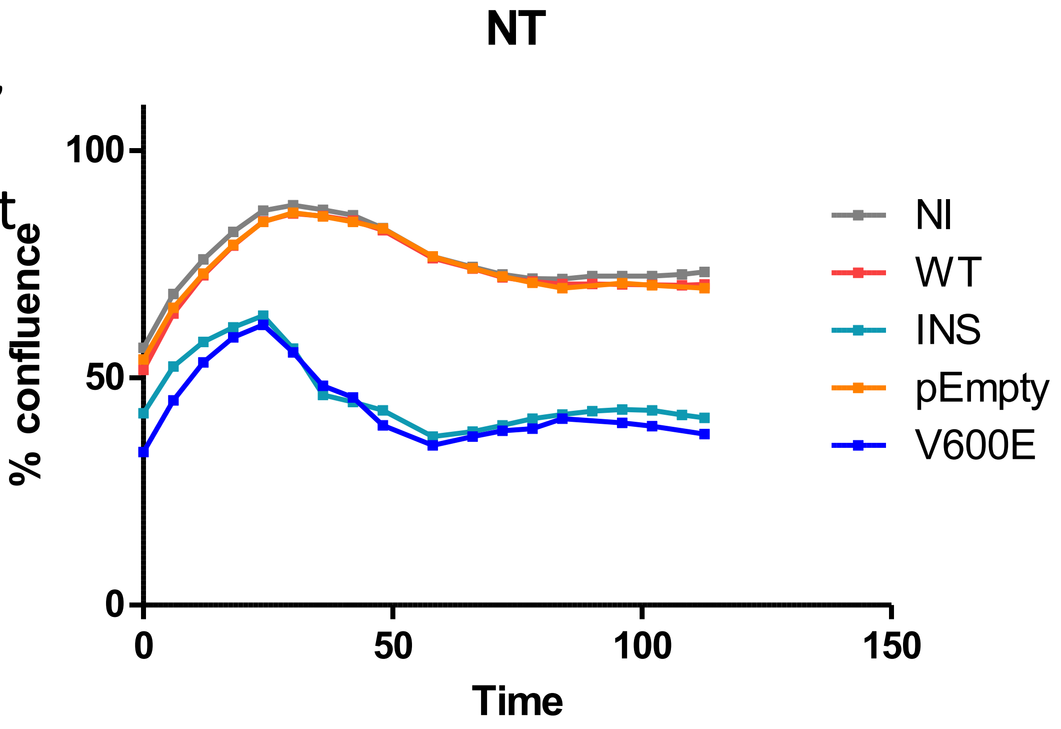

Proliferation with drugs

- Sorafenib impacts the non-mutant cell lines and lowers their proliferation.
- As expected the mutant cell lines are less affected by it.

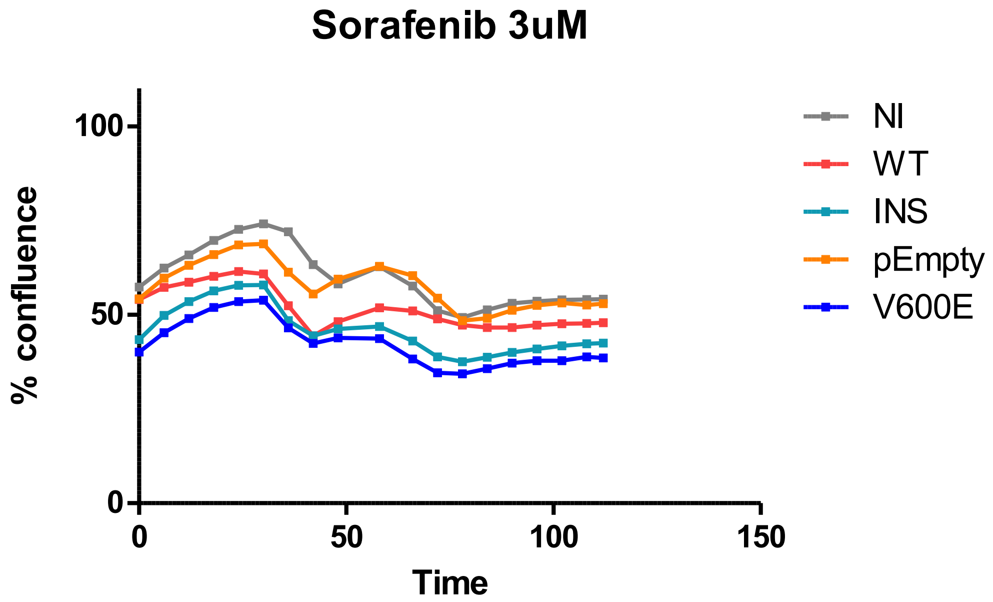

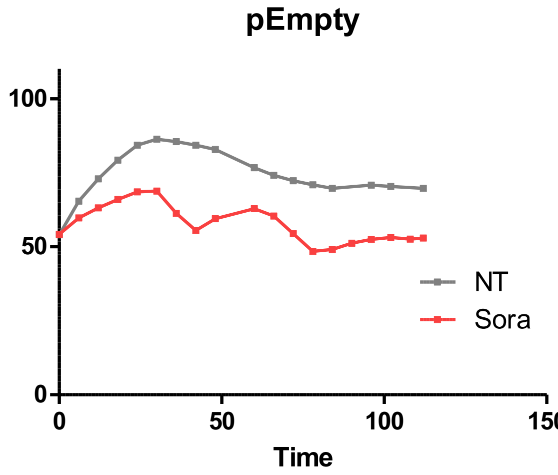

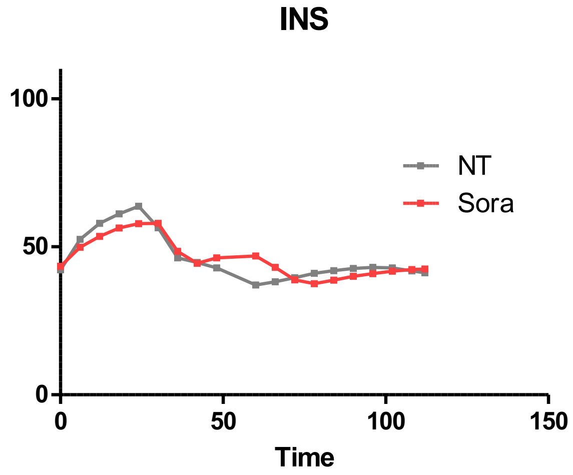

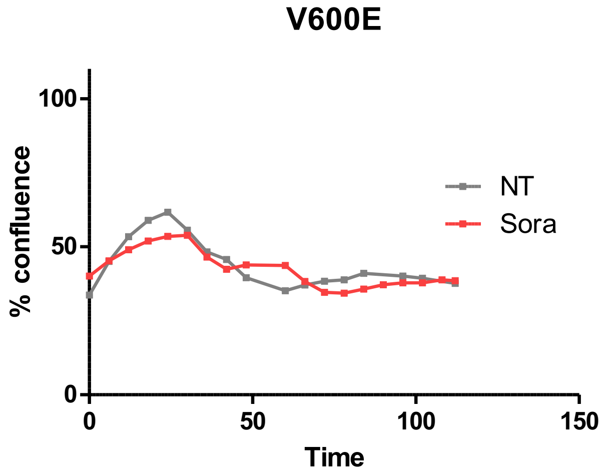

Proliferation with drugs

- Vemu impacts the mutant cell lines.
- While it does not impact the Empty vector or the WT.

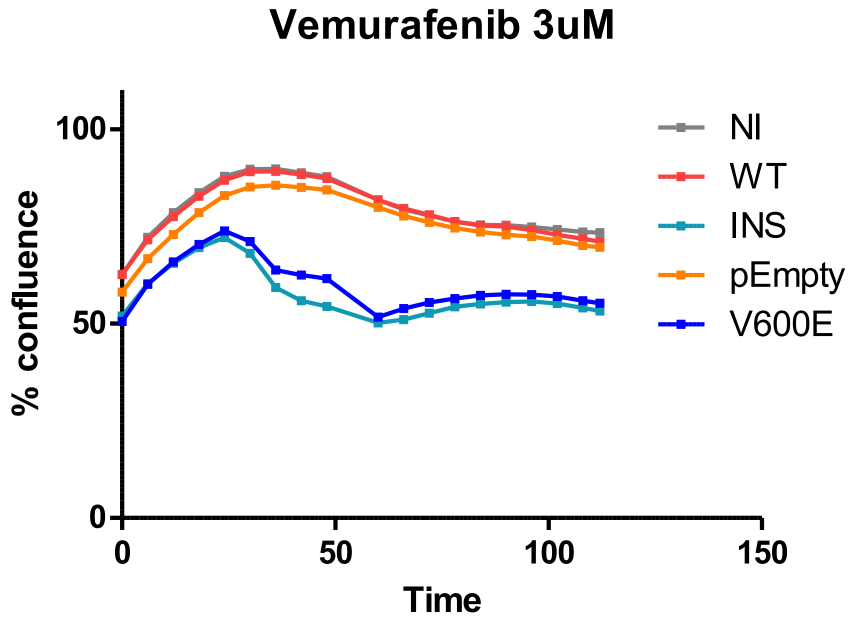

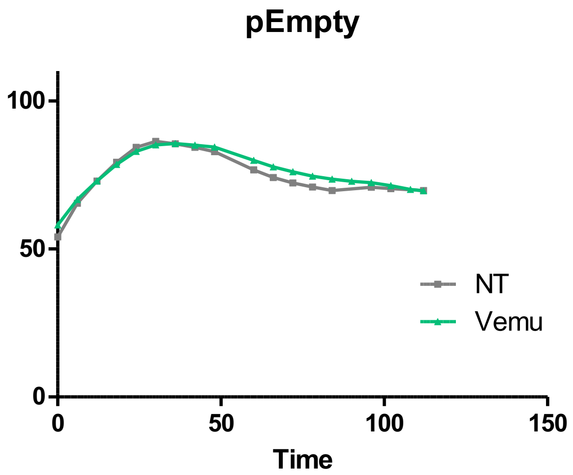

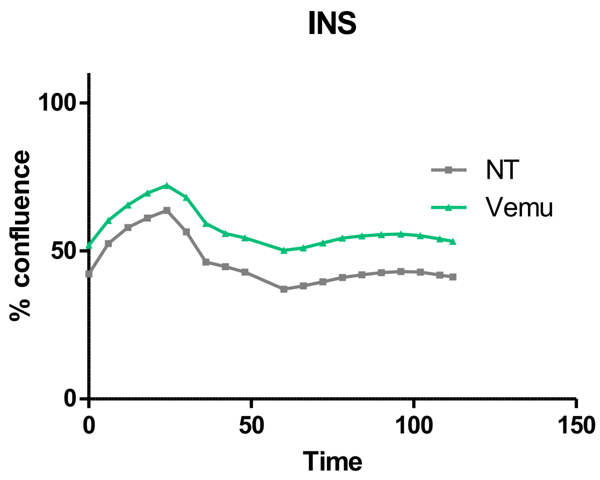

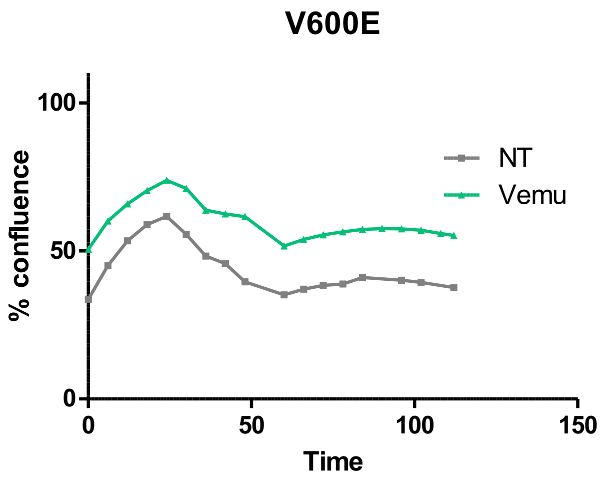

Proliferation with drugs

- Treatment with trame 1uM or 3uM does not affect the Empty vector or WT cell lines.
- But it change the comportement of the mutant cell lines to a comportement closer to the pEmtpty cell lines

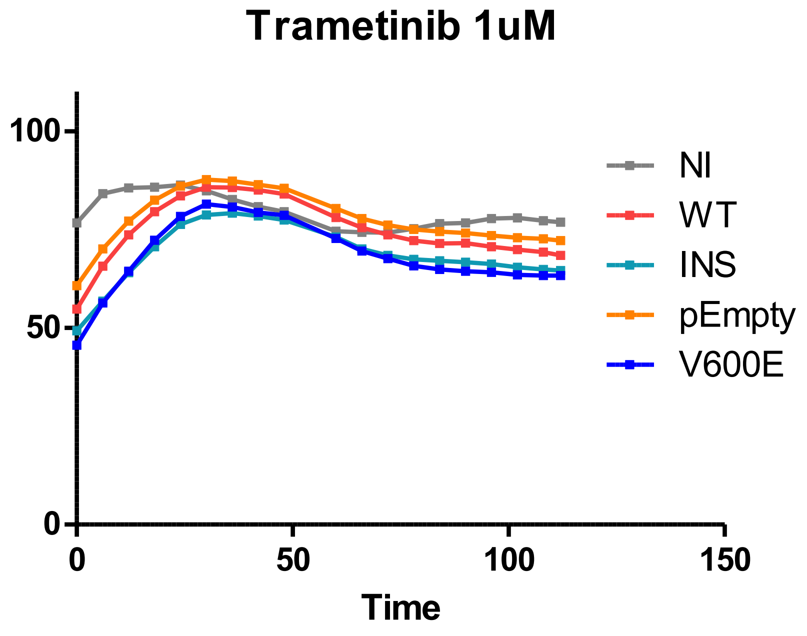

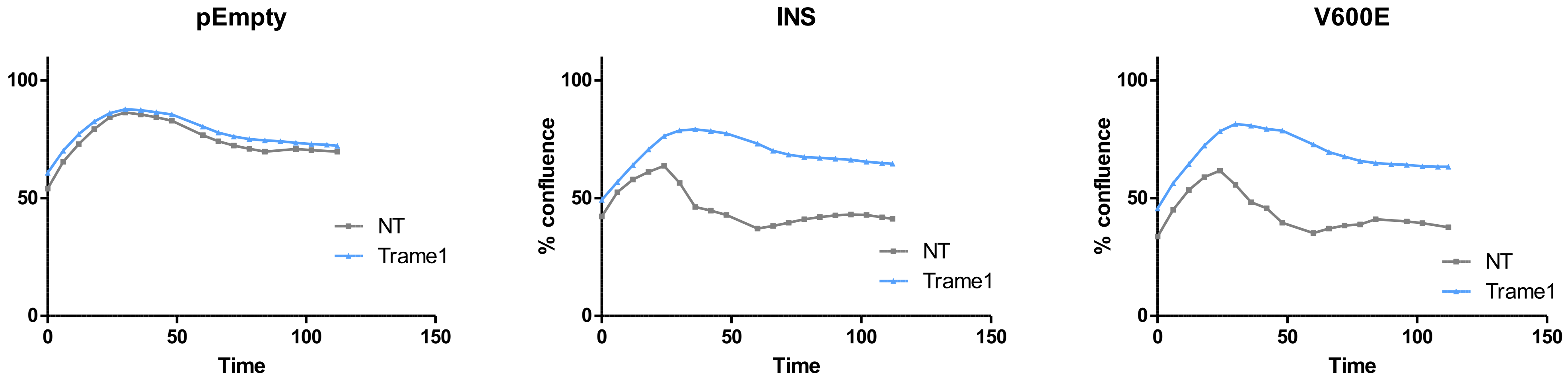

Proliferation with drugs

- With trame 3 uM all cell lines exhibit the same comportement.

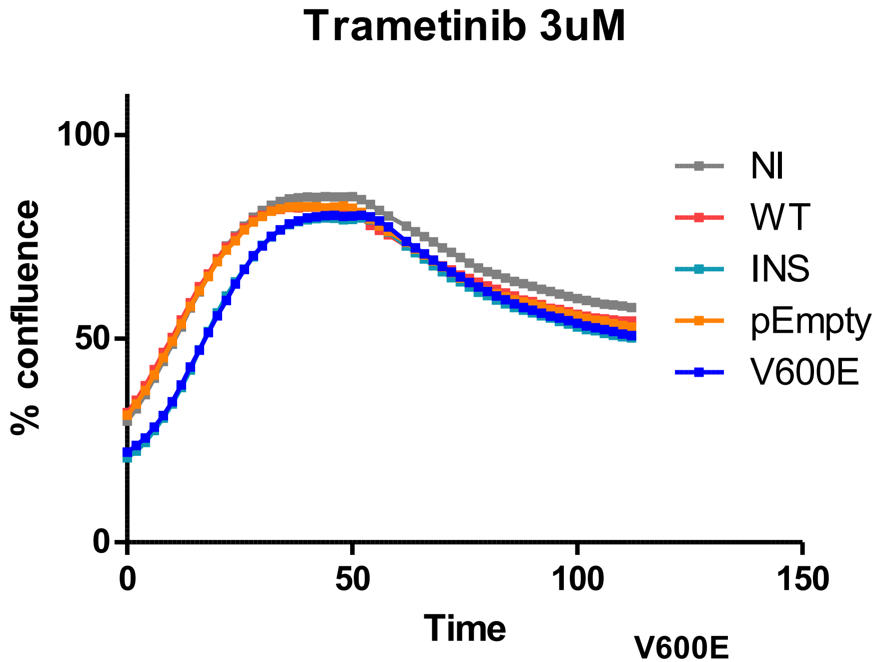

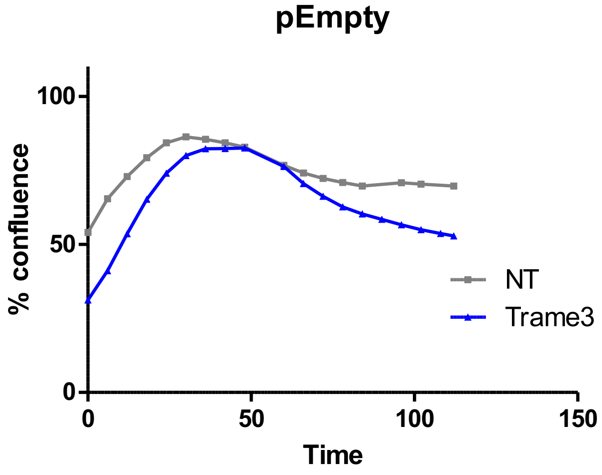

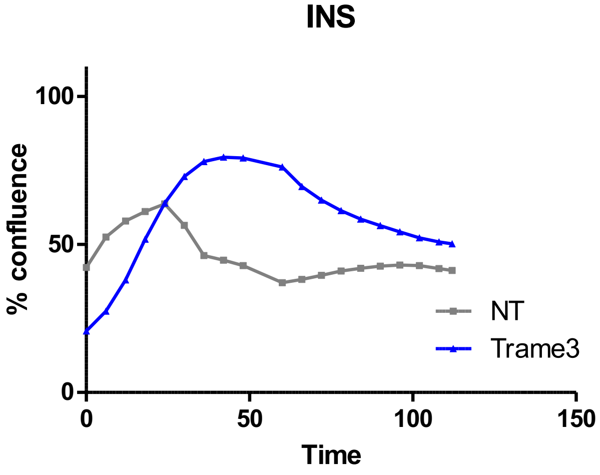

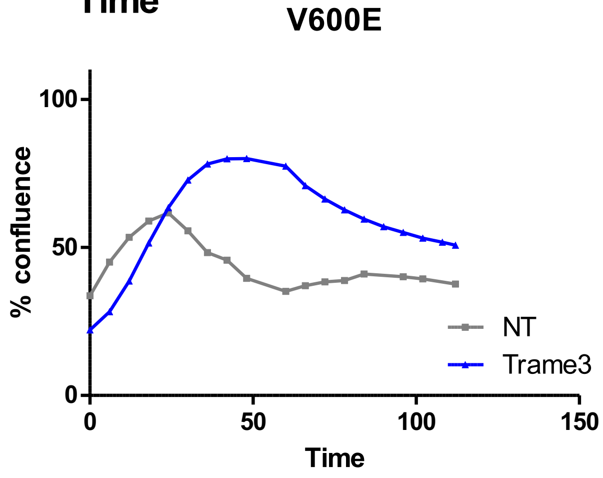

Supplement: Supplementary file 6 — Supp_Data_For_reviewer [file 41388_2018_623_MOESM6_ESM.pdf]
