# Supplementary material for: Recurrent somatic BRAF insertion (p.V504_R506dup): a tumor marker and a potential therapeutic target in pilocytic astrocytoma
Source: Oncogene. 2018 Dec 21;38(16):2994–3002. doi: 10.1038/s41388-018-0623-3 (PMC6484687; doi:10.1038/s41388-018-0623-3)
Supplement: Supplementary file 7 — Supp_Data_For_reviewer [file 41388_2018_623_MOESM7_ESM.pdf]

Index	Specimen label	Clinical Site #	Case #	DOB (mm/dd/yyyy)	Sex	Ethnicity (Race)	Clinical Diagnosis	Date of procurement	Anatomical Site	Tumor Location	Tissue Specification	Specimen Matrix
					male	caucasian	Astrocytoma		brain right temporal lobe	Primary	Tumor	Tissue
					male	caucasian	Astrocytoma		Blood	n/a	Normal	Blood

Index	Specimen Format	Container	Number of containers	Amount/per container	Unit	Type of Procurement	Histological description (Source)	Grade (source)	TNM Stage (T)	TNM Stage (N)	TNM Stage (M)	Treatment type	Component of treatment (Chemo / Horm Th Details)	Tumor cell %
	OCT	block	1	200	mg	surgery	Diffuse astrocytoma	2	n/a	n/a	n/a	none	n/a	80
	frozen	tube	1	4	ml	blood draw	n/a	n/a	n/a	n/a	n/a	none	n/a	n/a

ICD-O-3
Astrocytoma, diffuse 9400/3
Site: (R) Brain, Supratentorial C71.0
JtD 4/2/13

UUID:13BDC5C7-92EB-4FCD-ACAB-8005C2F099D4
TCGA-P5-A5EY-01A-PR
Redacted

Criteria [illegible] 12/19/12	Yes	No
Diagnosis Discrepancy		✓
Primary Tumor Site Discrepancy		✓
HIPAA Discrepancy		✓
Prior Malignancy History		✓
Dual/Synchronous Primary Noted		✓
Case is (circle): QUALIFIED / DISQUALIFIED		
Reviewer Initials BCH Date Reviewed: 12/19/12		
